# Employee voice behavior as a critical factor for organizational sustainability in the telecommunications industry

**DOI:** 10.1371/journal.pone.0238451

**Published:** 2020-09-03

**Authors:** Um-e-Rubbab, Sayyed Muhammad Mehdi Raza Naqvi

**Affiliations:** Capital University of Science and Technology, Islamabad, Pakistan; University Hospital Eriangen at Friedrich-Alexander-University Erlangen-Numberg, GERMANY

## Abstract

Organizational effectiveness is contingent upon employees’ contributions; however, the role of employee voice behavior as a critical component of employees’ contribution to the organization has not been sufficiently acknowledged. Based on proactive behavior theory, we present a model to investigate employee voice behavior as an underlying mechanism in the relationship between supervisor delegation and perceived workplace inclusion. Using the SEM (structural equation modeling) method, we test our model’s hypotheses with data from 271 employee-supervisor questionnaires administered in state-owned enterprises in the telecommunications industry. The results show that supervisor delegation is positively related to employees’ promotive and prohibitive voice behavior. Promotive voice significantly influences perceived workplace inclusion, but prohibitive voice behavior was not found to have any impact on perceived workplace inclusion. Moreover, both dimensions of voice behavior, i.e., promotive and prohibitive voice behavior, significantly mediate the relationship between supervisor delegation and perceived workplace inclusion.

## 1. Introduction

Work environments are changing in response to rapid innovation, competition, and long-term sustainability. The increasingly globalized work environment has encouraged organizations to continuously adapt, learn, and innovate for both long-term survival and better organizational performance [1]. Organizations’ external environments have become highly uncertain as a result of market competitiveness and deregulation policies, which have threatened organizational sustainability [2]. Increasingly complex workplace settings require employees to perform beyond their formal job descriptions and thus engage in extra-role behaviors [3]. Nowadays, organizations want to ensure their long-term viability in order to keep ahead of their competitors. It is impossible for organizations to remain competitive with workers who merely obey orders and do not contribute to the organization through feedback mechanisms. Instead, organizational sustainability is enhanced by employees who go beyond their formal job descriptions [4]. Thus, today’s dynamic and uncertain business environment has increased the importance of proactive behaviors for organizations’ long-term sustainability, change management, and adaptation [5–7]. One example of such proactive behavior is employee voice behavior. An organization is said to be more sustainable when its employees are highly involved in continuous change processes [8]. However, there is scant literature on the importance of employees as critical actors in change management processes [9].

Voice behavior is discretionary in nature and considered a form of proactive behavior [10, 11]. Proactive behaviors are future-oriented, extra-role behaviors that focus on making things happen [10]. This study investigates employee voice behavior as a way of meeting organizations’ need for long-term sustainability. Organizational sustainability requires highly engaged employees throughout the organization, which can be achieved by encouraging employees to engage in voice behavior related to organizational improvements [9]. Proactive behavior is considered crucially important for effective organizational functioning in an ever-changing business environment as it tends to challenge the status quo [12].

Voice behavior is of utmost importance for organizational innovation [13]. Organizations in which employees do not engage in voice behavior and withhold information tend to experience low employee engagement and motivation [14]. Voice is a mechanism through which employees can help their organization adjust to the current business environment and remain innovative [15]. However, voice behavior has received comparatively little attention in the management field. While proactive behavior theory has identified the mechanism through which voice takes place, there is scant research on the antecedents and consequences of employee voice behavior. Proactive behavior is triggered by certain motivational states and affect-related processes [16]. According to Parker et al.’s proactive behavior theory [16], a person modifies his/her behavior depending upon the outcome of this behavior in the past. Thus, if engaging in voice behavior results in less inclusion from others, a person's propensity to engage in voice behavior might decrease in the future. Consequently, if these less favorable outcomes make employees less likely to engage in voice behavior, organizations will be unable to prevent loss and harm and improve their functioning and long-term sustainability.

Research on voice took off in 1994, when Van Dyne and LePine [17] defined voice behavior as expressions of concern with the intention of improving the ways things are done in the organization and developed a scale to measure it. Moreover, they suggested that voice has a positive influence on organizational functioning, as it allows new ways of doing things to be identified and guides managers’ attention to solving critical issues related to existing practices. Voice, as the expression of constructive opinions about work-related issues, is considered an effective way for employees to show that they care about their workplace [15].

In contrast to previous research taking voice to be unidimensional, Liang and colleagues [15] distinguish between promotive and prohibitive voice behavior. Promotive voice involves suggestions to improve organizational processes. The motivation behind this type of voice is to make the organization a better place. In contrast, prohibitive voice involves stating one’s concerns about possibly troublesome policies, practices, and work behaviors [15]. Prohibitive voice is also essential, as it draws attention to previously undetected problems [15, 18, 19]. The motivation behind this type of voice is basically to move organizations away from problematic states [15, 19, 20]. Both dimensions of voice behavior are crucial for keeping the organization sustainable and competitive. However, much of the existing literature focuses more on the promotive dimension of voice, with less attention paid to the prohibitive dimension [18, 21, 19, 20].

In terms of predictors of voice behavior, studies have shown that specific personality characteristics, achievement striving, and voice climate may influence an employee’s tendency to engage in voice behavior [22–24]. Parker, Bindle, and Strauss [16] stated that contextual characteristics, including job design, the role of leadership, and workplace climate, play a role in predicting proactive behavior. Previous research on contextual characteristics as predictors of voice behavior has explored the role of job autonomy and job control [25], finding a U-shaped relationship between job control and voice behavior. Transformational and participative leadership styles have also been studied as predictors of proactive behavior [16]. Another positive leadership behavior which has gained attention lately is supervisor delegation, which is defined as managers’ tendency to delegate responsibility, power, and authority to people at lower levels of the organizational hierarchy [26]. This study seeks to examine the role of supervisor delegation as a factor that facilitates employee voice behavior. If workers perceive their superiors as receptive to their opinions and concerns and have a high-quality relationship with their superiors in the workplace, they will be more likely to engage in proactive behavior [17, 27]. Likewise, when employees have authority over their own tasks, they are more likely to speak up [28, 29]. Thus, supervisor delegation may motivate employees to engage in voice behavior.

Existing research on the consequences of voice behavior has shown that employees may use voice behavior to raise their status within the organization, for impression management, to achieve better performance evaluations as well as to enhance unit-level learning [18, 21, 19, 20]. In other words, employees display voice behavior in order to obtain benefits. One of the benefits employees hope to gain is workplace inclusion. The extant research on employee voice suggests that a majority of employees engage in voice behavior for the sake of getting equal opportunities, as voice helps them feel included in the organization [18–22]. Hence, we believe that workplace inclusion is an important outcome of promotive and prohibitive voice that has not received the attention it deserves from organizational behavior scholars [30]. There have been repeated calls to study its underexplored antecedents [31, 30]. Thus, the current study aims to fill this gap by testing promotive and prohibitive voice’s association with workplace inclusion.

**Fig 1:**
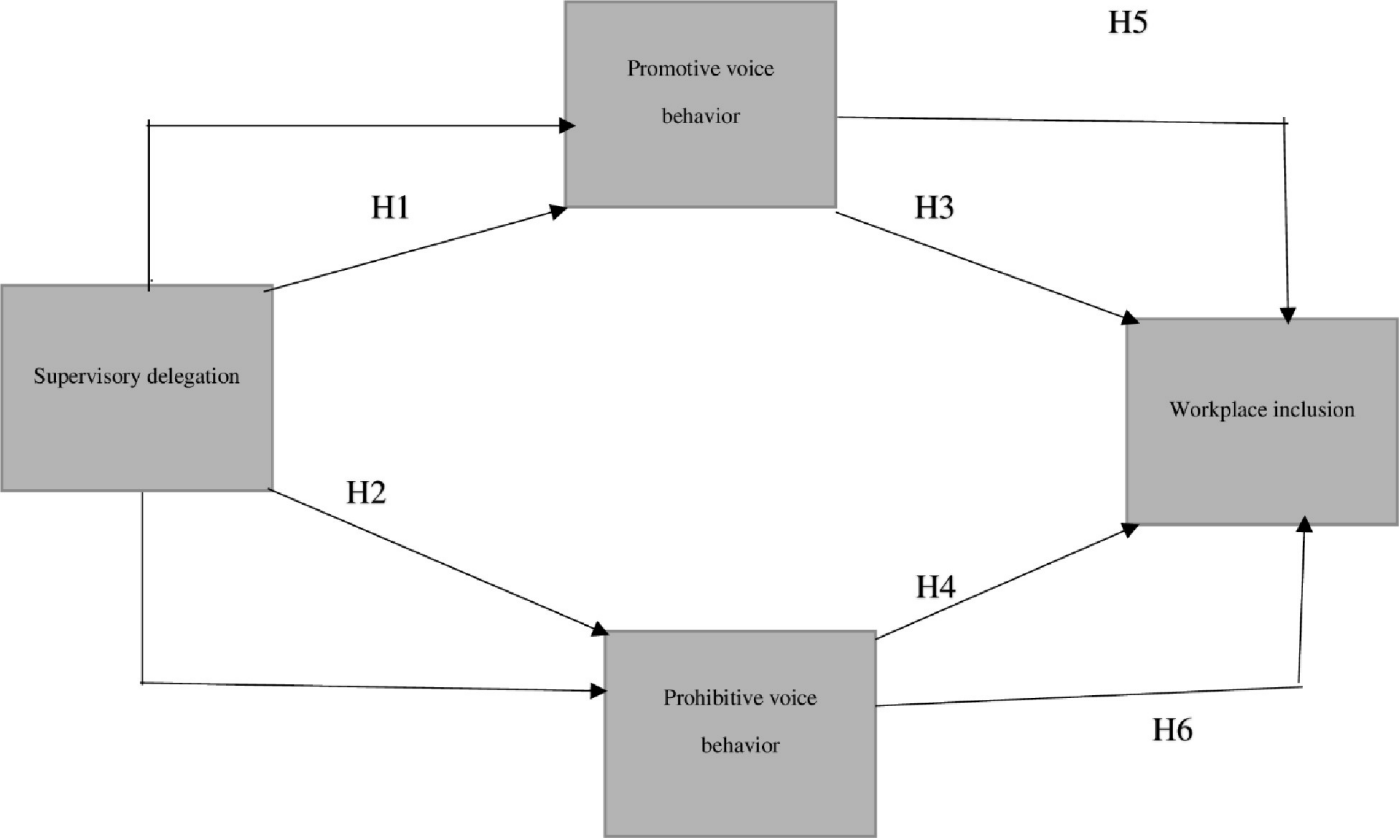
Conceptual representation of study variables.

Hence, this research extends previous studies on contextual antecedents of voice behavior by considering the role of an underexplored aspect, namely supervisor delegation. The proposed model is rooted in proactive behavior theory. The antecedents and outcomes of voice behavior addressed in this study are consistent with Parker, Bindl, and Strauss’ [16] argument that proactive behavior can be influenced by leaders' behavior. Hence, supervisor delegation is considered as a contextual antecedent of voice behavior, while perceived workplace inclusion is considered as an outcome. Fig 1 provides a conceptual depiction of the study variables.

### 1.1 Literature review

#### 1.1.1 Relationship between supervisor delegation and employee voice behavior

Supervisor delegation is defined as managers’ tendency to delegate responsibility, power, and authority to people at lower levels of the organizational hierarchy [26]. Delegation can enhance subordinates' decision-making skills and feelings of being valuable and trusted by the organization [26, 32]. Giving subordinates greater authority, discretion, and responsibility to make decisions is known as delegation [33]. Several benefits of supervisor delegation have been cited in the literature: higher-quality and quicker decisions, greater commitment to the organization and increased intrinsic motivation among subordinates [26], as well as better performance and job satisfaction [34, 26]. Delegating duties and responsibilities enables employees to speak out about what they really think is right and beneficial for the organization Moreover, lower-level employees may have a more profound level of understanding and expertise with respect to certain issues [35] because they are usually closer to the issues and customers. Thus, delegation can increase organizational efficiency. Delegation also decreases management overload and enhances subordinates’ leadership skills by providing them with an opportunity to exercise their decision-making skills [26]. Delegation is relevant to many topics of current interest in the management literature, such as employee involvement, empowerment, decentralization, and self-managed groups. When supervisors engage in delegation, employees become accountable for organizational sustainability, which encourages them to improve organizational processes and point out the flaws in the organization. Employees whose supervisors engage in delegation consider themselves more important and trusted [32]. Research suggests that when employees perceive that they are considered valuable by the organization, their performance improves and they experience higher motivation for work behaviors [18, 36].

Morrison and Milliken [37] point out that one reason employees do not engage in voice are organizational practices and policies that hinder employees’ involvement in decision-making and a lack of feedback mechanisms. Hence, we propose that delegation from managers to subordinates may encourage the latter to engage in voice behavior. In this way, managers should be able to exert a strong effect by creating favorable environments for promoting employees’ agency [38] in which responsibilities and authority are delegated to employees at lower levels of the organizational hierarchy. Hence, this study fills a gap in the existing research by investigating the effect of supervisor delegation on employees’ voice behavior.

Hypothesis 1: There is a positive and significant relationship between supervisor delegation and promotive voice behavior.
Hypothesis 2: There is a positive and significant relationship between supervisor delegation and prohibitive voice behavior.

#### 1.1.2 Relationship between employee voice behavior and perceived workplace inclusion

According to Maslow’s [39] hierarchy of needs, inclusion is considered a basic human need related to belonging. Everyone has a need to be included, recognized, and accepted by others at the workplace. Inclusion is defined as the extent to which employees consider themselves to be valued within their workgroup and results from experiencing treatment by others that satisfies their need for belongingness [40]. Perceived inclusion refers to the extent to which employees feel a part of organizational processes, which means that they feel connected to others at the workplace, believe that they have an influence and can participate in decision-making process, and have access to information [41–43]. Thus, higher perceived inclusion among employees should lead to higher organizational effectiveness because perceived inclusion influences employees’ sense of belongingness and attachment to the organization, which is ultimately beneficial for organizational effectiveness. However, research examining how managers respond to voice has been limited.

Promotive voice relates to improvements to existing work practices and procedures that may help the organization adapt to a dynamic environment [44, 17], whereas prohibitive voice relates to existing work practices that have the potential to harm the organization [15]. Hence, it makes sense to expect that managers appreciate and encourage promotive voice. In contrast, managers may discourage prohibitive voice because it can sound like a criticism of the organization. Promotive voice is considered more constructive in nature, so we expect managers to be able to identify employees’ good intentions when they engage in this form of voice. Consequently, promotive voice has a higher chance of being endorsed by management.

In contrast, prohibitive voice, which can involve pointing out a decline in the workgroup’s productivity due to problematic and unhealthy practices, is considered more challenging in nature [45]. Because prohibitive voice challenges the status quo [15, 21] and tends to point out unhealthy and problematic practices which are harmful to the organization in the long run, it is often considered a complaint. Existing research suggests that managers react poorly to employees who engage in voice for more person-centered reasons, and such employees experience increased retaliation by management [46].

Employees who engage in promotive voice are considered to be more effective by their supervisors than those who do not engage in promotive voice [15, 18, 19, 45]. Interestingly, however, employees who engage in prohibitive voice are considered less effective than those who do not engage in prohibitive voice [45]. Chamberlin et al. [45] further added that promotive voice, which concerns opportunities to improve the organization, is rewarded, whereas prohibitive voice, which protects the workplace from loss and damage, is disliked. Thus, different outcomes are expected depending on the nature of voice. Consequently, we propose:

Hypothesis 3: There is a positive and significant relationship between promotive voice behavior and perceived workplace inclusion.
Hypothesis 4: There is a negative and significant relationship between prohibitive voice behavior and perceived workplace inclusion

#### 1.1.3 Voice behavior as a mediator between supervisor delegation and perceived workplace inclusion

Morrison and Milliken [37] found that employees are usually reluctant to engage in voice when they fear that doing so would cost them heavily. Speaking up about an issue that management would prefer to ignore may cause a loss of relationships as well as the withholding of resources [47, 48]. Supervisors may feel insecure about employees’ input and voice and may therefore develop a negative perception of employees who frequently engage in voice behavior [18, 49]. Given that both types of voice tend to question managers’ decisions [50], voice behavior tends to disturb an employee’s interpersonal relationship with the manager and also has the potential to annoy senior management [51, 21].

Because voice behavior focuses a critical eye on current working conditions and thus has the potential to challenge the status quo [16], it is often resisted by others at the workplace. Thus, it is argued that employees who engage in voice typically experience both positive and negative reactions by others. Taking up this issue, this study seeks to extend the literature on voice by analyzing the mediating effect of two different types of voice behaviors on perceived workplace inclusion. Due to the positive nature of promotive voice, it usually gets a positive response and is welcomed, whereas prohibitive voice is not encouraged by organizations as it aims to identify loopholes in the organizational structure, processes or operations [45].

As the previous discussion makes clear, employees who are recipients of delegation by senior management will tend to engage in voice behavior, which consequently serves as an underlying mechanism for the relationship between supervisor delegation and employees’ perceptions of workplace inclusion. When employees engage in promotive voice behavior, they are included by others at the workplace, whereas when they engage in prohibitive voice behavior, they are less likely to be included by others in discussions, chitchat, and social interactions. Thus, it is argued that promotive voice behavior and prohibitive voice behavior mediate the relationship between supervisor delegation and perceived workplace inclusion. Consequently, we hypothesize that:

Hypothesis 5: Promotive voice behavior mediates the relationship between supervisor delegation and perceived workplace inclusion.
Hypothesis 6: Prohibitive voice behavior mediates the relationship between supervisor delegation and perceived workplace inclusion.

## 2. Materials and methods

### 2.1 Ethics approval

Ethical approval was obtained from the Departmental Ethics Committee of the Capital University of Science and Technology, Islamabad. The study procedure included no unethical behaviors. Our study did not involve clinical trials or experiments with human beings or animals. The content of the questionnaires had no sensitive information requirement. Participants were informed of the study objectives and consent was obtained verbally before the start of the study. Participation in the study was voluntary, and only individuals who were willing to give their consent participated. All participants were assured of their anonymity and confidentiality.

### 2.2 Procedure

The current study used a non-probability convenience sampling technique. The researchers used professional and personal contacts for data collection purposes. A cover letter was attached to the questionnaire in which the study’s purpose was explained and instructions were provided. Participation in the survey took place on a voluntary basis and participants’ confidentiality was strictly maintained. The current study’s data is multi-source, as it is taken from supervisors as well as employees. Two separate questionnaires were prepared for supervisors and employees, respectively. Supervisors were asked to rate their subordinates’ voice behavior. For this purpose, they were instructed to enter the full name of the employee for which they were providing responses. Employees’ responses were not shared with their supervisors, and they were assigned secret codes to maintain anonymity. Afterwards, the supervisor’s response was matched with the corresponding employee’s response using the already assigned code. Ethical issues were fully considered during the data collection process. Participants consent was obtained verbally before the start of the study. The supervisors’ and employees’ responses were kept confidential and anonymity was maintained.

### 2.3 Sample

Study respondents were recruited from state-owned enterprises in the telecommunications industry. There is intense competition in the telecommunications sector, which has led organizations to encourage their employees to engage in voice behavior, and constructive suggestions to help foster sustainable development are appreciated. We employed multisource questionnaires, which were filled out by both supervisors and their subordinates. The employee questionnaire asked participants about supervisor delegation and perceived workplace inclusion. The supervisor questionnaire asked about employees' engagement in voice behavior (promotive voice and prohibitive voice). A total of 299 employees and 61 supervisors returned the distributed questionnaires. After scrutiny, the total sample consisted of 271 valid responses, comprising 271 subordinates and 51 supervisors.

Table 1 provides demographic information about the employees. The sample consisted of 45% male and 55% female employees. The respondents were classified into five major age groups: 13% age 25–30, 24% age 31–35, 19% age 36–40, 26% age 41–50, and 18% above age 50. Respondents were categorized into three types of educational backgrounds: 34% with bachelor’s degrees, 44% with master’s degrees, and 22% with MS degrees. The respondents’ organizational tenure was also divided into five categories: 6% employees with less than a year of experience at the organization, 16% with 1–3 years, 19% with 4–7 years, 34% with 8–10 years, and 26% with more than ten years.

**Table 1:** Employee sample characteristics.

	Frequency	Percentage
Gender		
Male	122	45%
Female	149	55%
Age		
25–30	36	13%
31–35	66	24%
36–40	50	19%
41–50	69	26%
Above 50	50	18%
Educational level		
Bachelor’s	91	34%
Master’s	119	44%
MS	61	22%
Organizational tenure		
Less than a year	16	6%
1–3 years	45	16%
4–7 years	50	19%
8–10 years	90	33%
More than ten years	70	26%

### 2.4 Measures

The scales used in this study were taken from existing studies. A 5-point Likert scale was used to measure all constructs, with 5 = strongly agree and 1 = strongly disagree.

#### 2.4.1 Supervisor delegation

Supervisor delegation was measured with a 6-item scale developed by Yukl, Wall, and Lepsinger [52]. Sample items include “My supervisor gives me areas where I decide on my own, after first getting information from him/her” and “My supervisor lets me make decisions by myself, without consulting with him/her.”

#### 2.4.2 Promotive voice behavior

Promotive voice behavior was measured using Liang et al.’s [15] five-item scale. Sample items include “He/she proactively develops and makes suggestions for issues that may influence the unit” and “He/she raises suggestions to improve the unit’s working procedure.”

#### 2.4.3 Prohibitive voice behavior

Prohibitive voice behavior was also measured using Liang et al.’s [15] five-item scale. Sample items included “He/she dares to voice out opinions on things that might influence efficiency in the work unit, even if that would embarrass others” and “He/she advises other colleagues against undesirable behaviors that would hamper job performance.”

#### 2.4.4 Perceived workplace inclusion

Perceived workplace inclusion was measured using a ten-item scale developed by Mor, Barak and Cherin [53]. This scale measures the extent to which employees consider themselves part of important organizational processes such as access to information, connectedness to co-workers, and workgroup engagement. Sample items include “My judgment is respected by members of the workgroup” and “People in the workgroup listen to what I say.”

### 2.5 Covariates

The covariates used in the study were the supervisor’s and subordinates’ age, gender, education, and organizational tenure. A one-way ANOVA was performed to check the covariates’ impact on the study variables. None of the covariates were found to significantly influence the study variables. ANOVA is a statistical method that is used to analyze the impact of one or more nominal variables treated as independent variables on dependent variables [54]. ANOVA has also been performed in several existing studies [55–57]. The one-way ANOVA results showed that the F-statistic for gender was insignificant across supervisor delegation, (F = .42, p>.05), promotive voice behavior (F = .03, p>.05), prohibitive voice behavior (F = .42, p>.05) and perceived workplace inclusion (F = .32. p>.05).

The F-statistic for age was insignificant across supervisor delegation, (F = .87, p>.05), promotive voice behavior (F = .15, p>.05), prohibitive voice behavior (F = .14, p>.05) and perceived workplace inclusion (F = 1.19. p>.05).

The F-statistic for educational qualifications was insignificant across supervisor delegation, (F = .92, p>.05), promotive voice behavior (F = .89, p>.05), prohibitive voice behavior (F = 1.7, p>.05) and perceived workplace inclusion (F = 1.2. p>.05).

The F-statistic for experience was insignificant across supervisor delegation, (F = .95, p>.05), promotive voice behavior (F = 1.7, p>.05), prohibitive voice behavior (F = .61, p>.05) and perceived workplace inclusion (F = 1.7. p>.05).

## 3. Data analysis

The current study used SPSS and AMOS for data analysis purposes. The correlations and reliability were tested using SPSS, whereas confirmatory factor analysis and hypothesis testing were conducted with AMOS. The reason for choosing AMOS to test the hypotheses is because it is robust as compared to SPSS because it offers confirmatory factor analysis as well as multivariate path analysis with latent variables [58–60]. A confirmatory factor analysis examines the model fit and interrelationships among variables based on the theorized model. As suggested by previous researchers, we used multiple indices to test model fit within CFA [61], such as a relative chi square value less than 3, which represents an overall model fit index [61,62]; comparative fit index (CFI), which is considered to have excellent fit at values equal to or greater than .95 [63]; Tucker-Lewis index (TLI), with a threshold of .90 or above [64], and root mean square error of approximation (RMSEA), with a threshold of .08 - .05 for adequate fit [65]. Hypotheses proposing direct and mediated effects were tested using multivariate path analysis with bootstrapping and a 95 percent confident interval, as suggested by a number of studies [66].

### 3.1 Results

We conducted confirmatory factor analysis for all variables under study, namely supervisor delegation, promotive voice, prohibitive voice and perceived workplace inclusion. The model fit indices for our four-factor model satisfied the threshold criteria [67], with relative chi square (CMIN/df) = 1.121, IFI (incremental fit index) = 0.97, TLI (Tucker-Lewis index) = 0.97, CFI (comparative fit index) = 0.97, RMSEA (root mean square error of approximation) = 0.02. These results indicate an adequate model fit. Results of confirmatory factor analysis are shown in Table 2.

**Table 2:** Confirmatory factor analysis.

Model	CMIN/df	IFI	TLI	CFI	RMSEA
Proposed 4-factor model	1.121	.97	.97	.97	.02

The model fitness indices for our hypothesized SEM model satisfied the threshold criteria [67], with relative chi square (CMIN/df) = 1.182, IFI (incremental fit index) = 0.96; TLI (Tucker-Lewis index) = 0.96; CFI (comparative fit index) = 0.96, RMSEA (root mean square error of approximation) = 0.02. These results indicate an adequate model fit.

### 3.2 Descriptive statistics and correlation analysis

Descriptive statistics, including the mean values and standard deviations of the variables used in the study as well as the correlation coefficients found between variables, are shown in Table 3. As can be seen in Table 3, supervisor delegation is significantly and positively related to promotive voice behavior (r = .40**, p < .01); hence, H1 is preliminarily supported. Supervisor delegation is also significantly and positively related to prohibitive voice behavior (r = .42**, p < .01); thus, H2 is also preliminarily supported. Promotive voice behavior is significantly and positively correlated with perceived workplace inclusion (r = .40**, p < .01); thus, H3 is preliminarily supported. Finally, prohibitive voice behavior is not significantly correlated with perceived workplace inclusion (r = .11, p > .01); thus, H4 was is not supported.

**Table 3:** Descriptive statistical analysis, reliability analysis, and correlation coefficient matrix.

	Mean	S.D.	1	2	3	4	5	6	7	8
Gender										
Age			.01							
Education			.00	-.28**						
Tenure			-.02	-.24**	.24**					
SD	3.3	.77	-.04	-.01	.08	.11	(.78)			
PMV	3.4	.72	.01	.01	.02	.14*	.40**	(.70)		
PHV	3.2	.81	-.01	.00	-.02	-.00	.42**	.40**	(.74)	
PWIN	3.1	.81	.03	-.03	.09	.11	.07	.40**	.11	(.84)

* p < 0.05

** p < 0.01. n = 271, reliabilities are given in parentheses. SD = supervisor delegation; PMV = promotive voice behavior; PHV = prohibitive voice behavior, PWIN = perceived workplace inclusion.

### 3.3 Hypothesis testing

The results of hypothesis testing for direct and indirect effects are shown in Table 4. Supervisor delegation was positively related to promotive voice (β = 0.55***, S.E = .10, p < .001), as well as to prohibitive voice (β = 0.59***, S.E = .10, p < .001). Thus, Hypothesis 1 and 2 are supported.

**Table 4:** Hypothesis testing.

Hypothesis	path	β	S.E.	C.R.	p
1	SD→PMV	.55	.10	5.1	***
2	SD→PHV	.59	.10	5.3	***
3	PMV→PWIN	.57	.11	5.2	***
4	PHV→PWIN	-.16	.09	1.7	.08
Indirect Effect					
		Β	p	LLCI	ULCI
5	SD→PMV→PWIN	.53	.001	.35	.79
6	SD→PHV→PWIN	.56	.001	.37	.81

SD = supervisor delegation; PMV = promotive voice behavior; PHV = prohibitive voice behavior, PWIN = perceived workplace inclusion. Bootstrapping sample size = 2000. LLCI = lower limit of confidence interval; ULCI = upper limit of confidence interval, N = 271.

Hypothesis 3 and 4 proposed a positive relation between promotive voice and perceived workplace inclusion as well as prohibitive voice and perceived workplace inclusion. As shown in Table 4, promotive voice was positively associated with perceived workplace inclusion (β = 0.57***, S.E = .11, p < .001); however, prohibitive voice was not significantly associated with perceived workplace inclusion (β = -0.16, S.E = .09, p > .08), thus supporting Hypothesis 3 but not supporting Hypothesis 4. Fig B in S1 Appendix shows the path diagram of direct effects between supervisor delegation, promotive voice, prohibitive voice and perceived workplace inclusion.

We examined the indirect effects proposed in Hypotheses 5 and 6 through bootstrapping with 95 percent confidence intervals. Hypothesis 5 stated that promotive vice behavior mediates the relationship between supervisor delegation and perceived workplace inclusion. The indirect effect was significant, and the upper and lower limits of the confidence interval did not include zero, hence proving that the indirect effect is significantly different from zero (β = 0.53, with 2000 bootstrapped samples, LL = .35, UL = .79, and p < .001) and supporting Hypothesis 5.

Similarly, Hypothesis 6 stated that prohibitive voice behavior mediates the relationship between supervisor delegation and perceived workplace inclusion. Although our mediator was not significantly associated with the outcome variable, we proceeded to examine the indirect effect of supervisor delegation on workplace inclusion via prohibitive voice. It is possible that the indirect effect turns out to be significant even if the independent variable to mediator (a path) or mediator to dependent variable (b path) is non-significant [66, 68–70]. Therefore, we conducted a bootstrapped test for the indirect effect, as it is suggested to examine mediation by quantifying the indirect effect alongside the inferential statistical test [66, 68, 69]. The results showed that the upper and lower limits of the confidence interval for the indirect effect of prohibitive voice behavior were significantly different from zero (β = 0.56, with 2000 bootstrapped samples, LL = .37, UL = .81, and p < .001), supporting Hypothesis 6.

## 4. Discussion

This paper investigated both dimensions of voice behavior, i.e., promotive voice behavior and prohibitive voice behavior, as outcomes of supervisor delegation and predictors of perceived workplace inclusion based on proactive behavior theory [16].

Our first hypothesis was supported, as supervisor delegation was positively related to employees’ promotive voice behavior. The results of the study confirmed that when supervisors engage in delegation, it enhances employees’ tendency to engage in promotive voice behavior. Thus, employees are encouraged to engage in promotive voice behavior when their supervisors trust them enough to delegate tasks. Promotive voice involves generating ideas and raising concerns about improving the organization, its practices and ways of doing things [15]. Our findings are consistent with previous studies that the supervisor plays a crucial role in generating employee voice [71, 72, 36]. Our second hypothesis was also supported, as supervisor delegation was positively related to employees’ engagement in prohibitive voice behavior. Our study’s findings are thus consistent with previous studies [71, 72, 36]. Delegation is a type of empowering behavior in which leaders share power with followers by engaging them in the decision-making process, thus showing trust [73–75, 76], which enhances their likelihood of engaging in voice behavior.

Our third hypothesis was supported, as promotive voice behavior was positively related to perceived workplace inclusion. Promotive voice behavior is more suggestion-oriented, and employees engaging in this type of behavior are liked and positively appraised by senior management [17, 77], thus enhancing their chances of experiencing perceived workplace inclusion. Voice behavior results from a process of analyzing the problematic situation and coming up with solution-oriented suggestions. If the resulting suggestion, i.e. voice behavior, is considered as a complaint or criticism, it may negatively influence relationships and create an unfavorable impression of the employee [37, 48, 78]. Thus, our fourth hypothesis posited a negative relationship between prohibitive voice behavior and perceived workplace inclusion; however, this was not supported by the results. We proposed this because prohibitive voice behavior is more challenging in nature, appraised poorly by managers, and employees engaging in prohibitive voice are interpreted as potential problem-creators [45] because they try to bring attention to problematic practices and procedures in the workplace [21, 79], thus decreasing the chances of inclusion. Our results did not support this hypothesis. Instead, it seems to depend upon how colleagues and coworkers interpret a person’s engagement in prohibitive voice behavior. Our fifth and sixth hypotheses concerned the mediating role of promotive voice behavior and prohibitive voice behavior in the relationship between supervisor delegation and perceived workplace inclusion. Both were supported. It is possible that when employees have their supervisor’s back, they feel supported and trusted to give feedback, and are subsequently acknowledged through perceived workplace inclusion. The importance of both dimensions of voice behavior cannot be denied, as they help the organization correct harmful practices that are crucial for organizations’ long-run effectiveness.

### 4.1 Contribution to theory building

Most existing research on voice behavior has taken voice as a unidimensional construct; in contrast, our paper attempts to study both dimensions of voice behavior, in accordance with Liang, Farh, and Farh [15]. Our study argues that the two dimensions have different characteristics, and are consequently interpreted differently by others. Although both promotive and prohibitive voice are challenging in nature, prohibitive voice is more aggressive and perceived as more threatening, and thus less welcomed by others in the workplace. According to proactive behavior theory, voice behavior is proactive in nature and needs to be triggered by certain situational antecedents [16]. Supervisor delegation serves as a contextual precursor to employees’ engagement in voice behavior, whereas perceived workplace inclusion is an outcome of voice behavior, meeting employees’ need to be included by others in the workplace. Perceived workplace inclusion serves to satisfy employees’ need for belongingness and social interaction [80]. Employees engage in voice behavior in order to enhance organizational sustainability by highlighting issues that may hamper organizational functioning. Thus, employees who engage in voice behavior may be seen by others as responsible colleagues and thus be included in workplace interactions.

### 4.2 Practical implications

The present study’s findings have numerous implications for managers not only in the telecommunication sector but also in other service sectors. Organizations are facing intense competition and challenges, and it has become difficult for organizations to offer financial and economic rewards to employees for their efforts [81]. Thus, management should cultivate an environment that fuels employee motivation. When employees feel that they have a voice and are heard, they realize their importance to the workplace. This should increase their tendency to engage in voice behavior. Voice behavior is risky in nature, requires effort and does not always receive approval, but when employees’ voice is appreciated, they feel noticed. Several studies have stated that employee voice behavior is critical for improved functioning, growth, error detection and organizational survival [5, 6, 7].

The current study suggests that managers should cultivate a workplace environment which is conducive for employees to feel confident engaging in voice behavior and raising concerns for improved organizational functioning. When managers delegate tasks and responsibilities to their subordinates, it is a way of showing respect for employees’ worth. Managers find opportunities to praise employees’ talents by delegating tasks. Delegation refers to the process of empowering employees so that they can perform their tasks independently. Thus, employees’ engagement in voice behavior can be triggered by supervisors themselves by delegating tasks, and this voice behavior in turn enhances employees’ perception of workplace inclusion. Perceived workplace inclusion enhances and boosts employee’s confidence in themselves [82]. Thus, managers need to design the workplace in a way that gives more autonomy to employees and in which participation in decision-making involves less risk.

This study’s findings are significant because most studies on voice behavior are conducted in the Western context, which is characterized by low power distance. In contrast, this study was conducted in a context that is high on power distance. Even when senior management engages in delegation and empowers employees, acknowledgment of employees’ voice varies. Organizations need to appreciate and retain more proactive workers who engage in voice behavior. Sustainable human capital has the potential to enhance organizational productivity [83]. Nevertheless, employees have to think about the degree of freedom they are given to engage in voice behavior. Voice behavior has the potential to challenge the status quo. Thus, employees in high power-distance cultures may wish to think twice before engaging in either dimension of voice behavior because they need to evaluate many other factors within the organization. While voice behavior can be rewarding for employees, the last thing they want is to hamper their relationship with senior management.

### 4.3 Limitations and future research directions

The current study has several limitations. The study tested the theory of pro-active motivation, i.e. a Western-based theory, with constructs in an Asian context. This reduces the external validity of the findings, because the data sample was also drawn only from Pakistan. Future studies should replicate this research in a context outside Pakistan. Secondly, research on employee voice behavior needs to be conducted with more caution and considering cultural differences [84], such as Pakistan’s high level of power distance [85].

The current study investigated direct and mediating effects between constructs. Future studies should investigate other mediating mechanisms that predict voice behavior as well as other possible outcomes of employee voice behavior. Applying proactive motivation theory, future researchers should examine other dispositional and leadership antecedents responsible for voice behavior. Furthermore, future studies should develop and test models with multilevel effects due to the nature of leadership-related antecedents, which can account for shared variance within members of a given group as well as variance between groups. In this regard, we also suggest that future studies consider supervisor delegation as a group-level phenomenon and examine its cross-level and multilevel effects.

We suggest that future researchers also examine the situational factors that shape voice behavior, such as a culture that is more conducive of voice behavior, as suppressing the voice mechanism can be detrimental for organizations’ long-term effectiveness. Effective organizational practices are essential for remaining competitive and profitable [86]. Employee voice behavior is one such sustainable practice. Future research may identify factors such as voice climate, voice efficacy, and voice safety as possible moderators of the proposed relationships.

### 4.4 Conclusion

Organizations strive to make their employees feel included, and supervisor delegation can help to achieve this objective. Supervisors must delegate authority to the employee in order to promote employee voice so that they feel included. Organizations need to respond to uncertain environments with efficiency and effectiveness [86], and this is not possible without employees’ active involvement in the form of voice behavior. Hence, in order to fully capitalize on the effects of voice behavior for organizational effectiveness, managers need to acknowledge both dimensions of voice behavior. Consequently, this study makes an important contribution by suggesting that managers can increase perceived workplace inclusion, as an important management practice, by delegating tasks and accepting employees’ voice behaviors. Perceived workplace inclusion is an effective HRM practice [40]. It tends to foster employees’ involvement and belongingness to the organization.

## Supporting information

S1 Appendix (DOCX)

S1 File (SAV)
