# Supplementary material for: Employee voice behavior as a critical factor for organizational sustainability in the telecommunications industry
Source: PLoS One. 2020 Sep 3;15(9):e0238451. doi: 10.1371/journal.pone.0238451 (PMC7470423; doi:10.1371/journal.pone.0238451)

**S1 Appendix**

**Figure A: Confirmatory Factor Analysis**

**
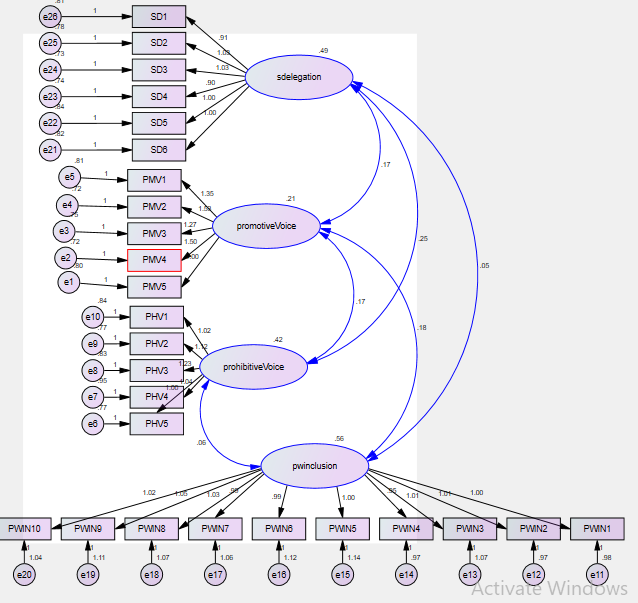
**

**Figure B: SEM Path Diagram for Latent Variables**


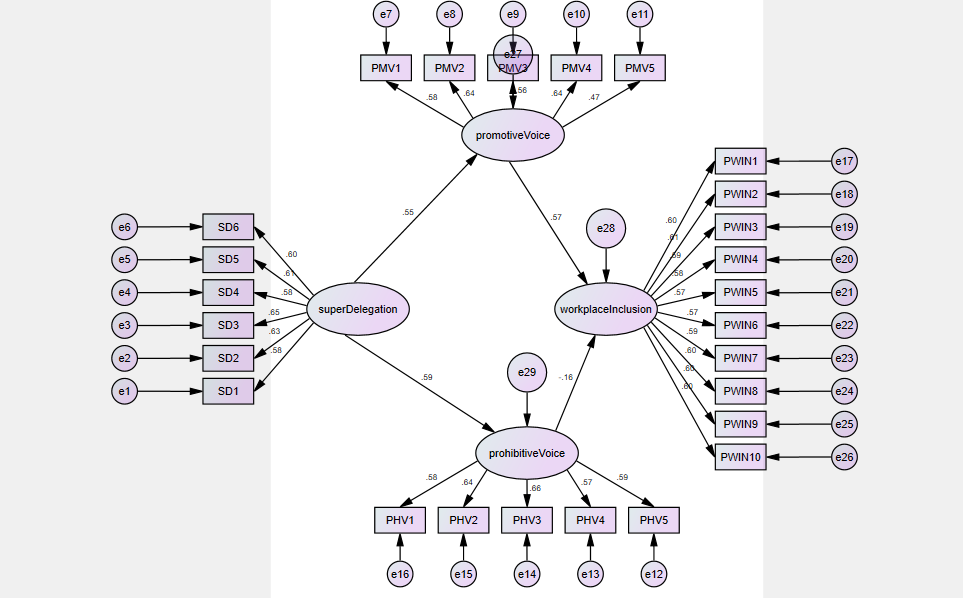

Supplement: S1 Appendix — (DOCX) [file pone.0238451.s001.docx]
